# Is serum 25‐hydroxyvitamin D deficiency a risk factor for the incidence of slow gait speed in older individuals? Evidence from the English longitudinal study of ageing

**DOI:** 10.1111/dom.16317

**Published:** 2025-03-13

**Authors:** Mariane Marques Luiz, Roberta de Oliveira Máximo, Aline Fernanda de Souza, Thales Batista de Souza, Sara Souza Lima, Leticia Coelho Silveira, Thaís Barros Pereira da Silva, Andrew Steptoe, Cesar de Oliveira, Tiago da Silva Alexandre

**Affiliations:** ^1^ Postgraduate programme in Physical Therapy Federal University of Sao Carlos Sao Carlos Brazil; ^2^ Postgraduate programme in Gerontology Federal University of Sao Carlos Sao Carlos Brazil; ^3^ Department of Behavioural Science and Health University College London London UK; ^4^ Department of Epidemiology and Public Health University College London London UK; ^5^ Gerontology Department Federal University of Sao Carlos Sao Carlos Brazil

**Keywords:** 25(OH)D, ageing, gait speed, mobility limitation, vitamin D

## Abstract

**Aims:**

Cross‐sectional studies demonstrate an association between low serum levels of vitamin D and slower gait speed in older individuals. However, longitudinal studies remain inconclusive. This study investigates whether vitamin D deficiency and insufficiency are risk factors for the incidence of slowness.

**Materials and Methods:**

A total of 2815 participants from the English Longitudinal Study of Ageing (ELSA), aged ≥60 years and with a baseline gait speed >0.8 m/s, were followed for six years. Baseline serum levels of vitamin D [25(OH)D] were categorized as “sufficiency” (>50 nmol/L), “insufficiency” (>30 and ≤50 nmol/L) or “deficiency” (≤30 nmol/L). Gait speed was reassessed at four and six years of follow‐up to identify incident cases of slowness (walking speed ≤0.8 m/s). A Poisson regression model, adjusted for sociodemographic, behavioural and clinical characteristics at baseline, was conducted to determine the association between serum 25(OH)D levels and the risk of slowness.

**Results:**

The incidence densities of slowness per 1000 person‐years were 67.4 (95% CI: 60.93–74.64) for sufficiency, 76.7 (95% CI: 68.30–86.22) for insufficiency and 90.7 (95% CI: 78.46–104.92) for deficiency. Serum 25(OH)D deficiency was associated with a 22% increase in the risk of slowness (IRR: 1.22; 95% CI: 1.01–1.49) compared with serum 25(OH)D sufficiency. No significant association was observed for serum 25(OH)D insufficiency.

**Conclusions:**

Serum 25(OH)D deficiency is a risk factor for the incidence of slowness in older individuals, suggesting that maintaining sufficient 25(OH)D levels could be a strategic approach to minimise long‐term mobility impairment.

## 1 INTRODUCTION

Mobility, defined as the ability to move independently within one's environment, is most accurately represented by walking capability.^1^, ^2^ Mobility limitation, characterized by poor gait performance and slow gait speed, represents the early stage of functional impairment, which can lead to a downward and progressive spiral of functional disability, particularly in older individuals.^3^, ^4^, ^5^

Although the gait speed decline with advancing age is multifactorial, some of the most prominent associated factors include comorbidities, musculoskeletal pain, falls, low muscle strength, visual impairment and low physical activity level.^6^ Moreover, many studies have investigated whether vitamin D [25‐hydroxyvitamin D or 25(OH)D] deficiency is associated with the decline in gait speed in older individuals.

Vitamin D deficiency affects approximately 15% of the world's older population.^7^ This phenomenon is linked to the lower bioavailability of the precursor of vitamin D (7‐dehydrocholesterol), which arises from the thinning of the skin and the decreased number of vitamin D receptors (VDRs) in cells during the ageing process.Consequently, older individuals have a reduced capacity for cutaneous synthesis and distribution of vitamin D to tissues.^8^

Given the presence of VDRs in muscle cells, vitamin D plays an essential role in muscle metabolism through genomic and non‐genomic pathways.^9^, ^10^ Vitamin D modulates calcium (Ca^2+^) influx into muscle cells, a process fundamental for muscle contraction and maintenance of muscle mass, strength and function.^9^, ^10^ Maintaining musculoskeletal integrity is vital for preserving mobility, especially the gait component.^11^ Given this, it is plausible that vitamin D deficiency may contribute to slowness in advanced age through its detrimental effects on muscle function. Therefore, cross‐sectional studies suggest a significant association between serum vitamin D deficiency and slower gait speed in older individuals.^12^, ^13^, ^14^, ^15^ However, longitudinal studies present conflicting data, which may arise from differences in sample characteristics and the methodologies employed.

For instance, in 988 individuals aged ≥77 years, no association was found between serum 25(OH)D deficiency (<50 nmol/L) and decline in gait speed in the 3‐m walk test after three years of follow‐up.^16^ Likewise, in 368 individuals aged 70 to 89, baseline serum 25(OH)D levels were not significantly associated with changes in the 400‐m walk test over 12 months.^17^ Furthermore, changes in 25(OH)D status over time were not associated with gait speed improvements.^17^ In contrast, a baseline association was found between serum 25(OH)D deficiency (<50 nmol/L) and low gait speed in the 20‐m and 400‐m walk tests in 2641 individuals aged 71 to 80 years. After four years, the association remained significant only for slow gait speed in the 400‐m walk test.^18^

Beyond these inconclusive results, no study to date has confirmed whether serum 25(OH)D levels are linked to the incidence of slow gait speed in older individuals without prior mobility limitations. Investigating this gap could enhance our understanding of the mechanisms inherent to the process of gait speed decline leading up to the onset of slowness. Therefore, the present study aimed to investigate whether there is an association between serum 25(OH)D insufficiency and deficiency and the risk of slowness in older individuals without previous mobility limitations.

## 2 MATERIALS AND METHODS

### 2.1 Study population

This study involved data from the English Longitudinal Study of Ageing (ELSA), a prospective panel study of community‐dwelling English subjects aged 50 years or older.^19^ The ELSA study was launched in 2002, with the sample consisting of individuals who participated in the Health Survey for England (HSE) in 1998, 1999 and 2001. The HSE recruits a nationally representative sample using a multi‐staged stratified random probability design.^20^ Since 2002, interviews have been conducted biennially through administering questionnaires. From 2004 onwards, home visits by nursing staff are carried out every four years for blood collection, performance tests and health examinations. Detailed descriptions of the study design and sampling procedures have been published previously.^19^

ELSA was conducted in accordance with the Declaration of Helsinki and received approval from the National Research Ethics Service (London Multicentre Research Ethics Committee [MREC/01/2/91]). All participants provided informed consent, and all studies and methods were carried out in compliance with the approved regulations and guidelines.

**FIGURE 1:**
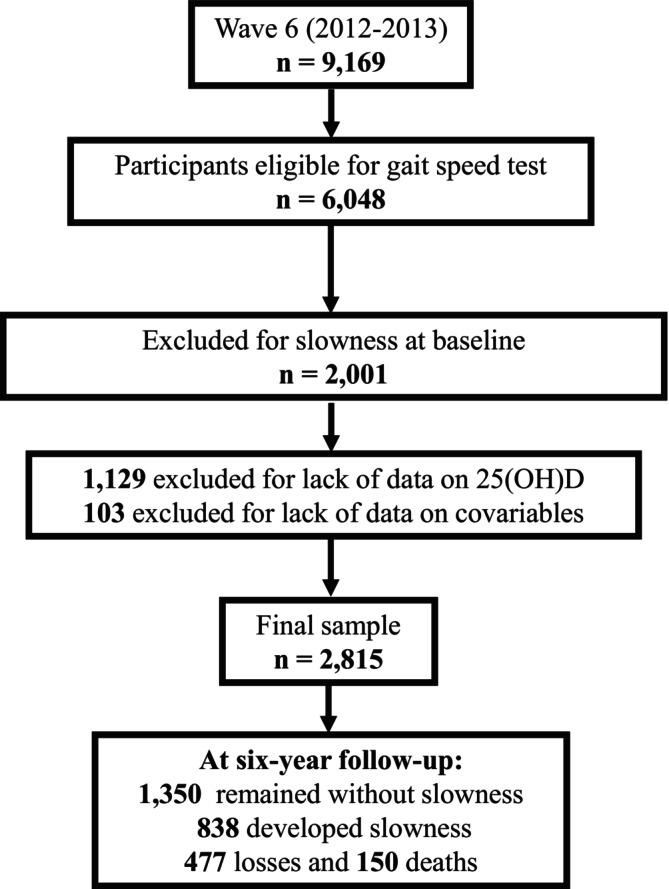
Flow chart depicting the sample selection process, detailing exclusions and the final analytical sample.

Wave six of the ELSA Study (2012/2013) served as the baseline for this study when serum 25(OH)D levels were first collected. Follow‐up data were collected in Wave eight (2016/2017) and Wave nine (2018/2019), totalling six years. Wave six comprised 9169 participants, of whom 6048 were eligible for the gait speed test, administered only to individuals aged ≥60 years. Of these, 2001 were excluded due to having slowness at baseline (gait speed ≤0.8 m/s), 1129 were excluded due to a lack of serum 25(OH)D levels data and 103 were excluded due to a lack of covariates data. The final analytical sample consisted of 2815 individuals (Figure 1).

### 2.2 Gait speed

Gait speed was measured by the time taken to walk 2.4 m at a habitual pace, either with or without a gait‐assistance device, on a flat surface in the participants' private homes. The adopted distance is commonly used and has been validated in previous studies.^3^, ^21^ Gait speed was assessed at baseline to identify and exclude individuals with slowness (gait speed ≤0.8 m/s).^22^ Gait speed was reassessed after four and six years of follow‐up, and those whose walking pace decreased to ≤0.8 m/s were considered incident cases of slowness.

### 2.3 Serum 25(OH)D levels

Blood samples to measure baseline serum 25(OH)D levels were analysed at the Royal Victoria Infirmary in the United Kingdom using the DiaSorin Liaison immunoassay, which has an analytical sensitivity of 7.5 nmol/L and a coefficient of variation ranging from 8.7% to 9.4%. All assays were conducted in duplicate, and the laboratory performing the analyses participated in the Vitamin D External Quality Assessment Schemes.^23^ Serum 25(OH)D status was classified according to the guidelines of the US Institute of Medicine: sufficiency (>50 nmol/L), insufficiency (>30 and ≤ 50 nmol/L) and deficiency (≤30 nmol/L).^24^

### 2.4 Control variables

Previous studies that analysed factors associated with serum 25(OH)D deficiency and the decline in gait speed were used to select control variables.^6^, ^25^ All control variables were collected at baseline.

Sociodemographic variables were gathered using self‐completion questionnaires. Age was categorized in 10‐year increments (60–69, 70–79 or ≥80 years), along with sex and race (white or non‐white). Marital status was defined as “with a conjugal life” (for married individuals or those in a stable relationship) and “without a conjugal life” (for single, divorced, separated or widowed individuals). Education was classified based on the English educational system (>13 years, 12–13 years or ≤11 years). Household wealth was determined by the value accumulated in savings accounts and investments, any property (excluding mortgages) or other assets, the worth of any active business and non‐debt physical wealth (artworks and jewellery). Total household wealth was categorized into quintiles.

Behavioural characteristics included self‐reported smoking status (non‐smoker, ex‐smoker or current smoker), frequency of alcohol consumption (rarely/never, often, daily or not reported) and levels of physical activity. Physical activity was assessed using the Physical Activity and Sedentary Behaviour Assessment Questionnaire from the HSE.^26^ The participants supplied information on the frequency (more than once per week, once per week, one to three times per month, and hardly ever or never) and intensity of the activity undertaken (vigorous, moderate or light). Participants were classified as active (engaging in vigorous or moderate physical activity more than once per week) or sedentary (engaging in vigorous or moderate physical activity once a week, one to three times per month, hardly ever or never, and any light physical activity).^27^

Clinical conditions were recorded based on individuals' self‐reports of a clinical diagnosis of systemic arterial hypertension, diabetes mellitus, cancer, lung disease, heart disease, stroke, osteoporosis, osteoarthritis and dementia.

The frequency of falls in the previous year (none, a single fall or recurring falls), and the occurrence of hip fracture were investigated. Visual perception was classified as “excellent or very good”, “good”, and “fair or poor”. Pain in the lower limbs (hip, knee or foot) while walking and back pain during walking were scored (0–10) and classified as “no pain,” “mild to moderate pain” (score: 1–5), or “intense to severe pain” (score: 6–10).^28^

Memory performance was assessed using a list of ten randomly selected words. After hearing the words, the participant was asked to recall as many as possible (immediate recall). Following a five‐minute interval, the participant was asked again to repeat as many words as possible (delayed recall). The total number of correctly recalled words in both parts of the test (range: 1–20) served as an indicator of memory performance, with higher scores reflecting better performance.^29^ Depressive symptoms were evaluated using the eight‐item *Centre for Epidemiologic Studies Depression Scale* (*CES‐D*), with a score of ≥4 points suggesting the presence of depressive symptoms.^30^

Waist circumference was measured using a metric tape at the midpoint between the lowest rib and upper margin of the iliac crest, with the participant standing, arms alongside the body, trunk bare and abdomen relaxed after expiration. Abdominal obesity was defined as waist circumference >102 cm for men and >88 cm for women.^31^ Grip strength was measured using a hand dynamometer (Smedley; range: 0–100 kg), with the participant standing, arms alongside the trunk and elbow flexed at 90°. The highest value obtained in the dominant hand among the three trials was considered in the analysis. Dynapenia was defined as grip strength <26 kg for men and <16 kg for women.^32^

Seasonal variation influences the incidence of solar radiation on the Earth's surface, affecting the cutaneous synthesis of vitamin D and its circulating levels.^33^ Therefore, the season of blood collection was used as a control variable (spring, summer, autumn and winter).^23^ Vitamin D supplementation and the use of carbamazepine, an anticonvulsant with the potential to reduce serum 25(OH)D levels, were also used as control variables.^34^

### 2.5 Statistical analysis

The sample characteristics were expressed as the mean, standard deviation and proportion. Differences in these characteristics according to baseline serum 25(OH)D status were analysed using the chi‐square test and analysis of variance (ANOVA) with Tukey's post hoc test.

For the calculation of incidence densities of slowness among those with serum 25(OH)D sufficiency, insufficiency and deficiency, the number of participants who developed slowness during the analysed period was taken as the numerator, whilst the total observation time of this population in each group was considered the denominator. Incidence densities per 1000 persons per year were presented with 95% confidence intervals (CIs).

Poisson regression models were employed to assess the association between serum 25(OH)D levels and the incidence of slowness, calculating incidence rate ratios (IRR) along with their respective 95% CIs. Slowness, defined as a gait speed of ≤0.8 m/s, was considered the outcome. Covariates with a p‐value <0.20 in the univariate analysis were chosen for inclusion in the multivariate model. Statistical significance in the final regression model was defined by a *p*‐value <0.05.^35^ In all analyses, the reference category was serum 25(OH)D sufficiency.

The goodness‐of‐fit for the Poisson regression models was assessed using the Hosmer–Lemeshow test. All analyses were conducted with the Stata 16® statistical program (StataCorp, College Station, TX, USA).

## 3 RESULTS

Among the 2815 individuals at baseline, the prevalence of serum 25(OH)D sufficiency, insufficiency and deficiency was 47.9%, 32.5% and 19.6%, respectively. Baseline characteristics of participants based on serum 25(OH)D levels are presented in Tables 1 and 2. The average age of participants was 68 years, and women comprised 52.2% of the sample. Most participants were individuals with white skin (98.4%), those in a marital relationship (72.9%), those with higher educational attainment (37.2%) and those in the highest wealth quintile (29%). In terms of behavioural characteristics, the majority of participants were ex‐smokers (52.8%), had a frequent alcohol intake (41.2%) and maintained an active lifestyle (78.3%). The most common clinical conditions included abdominal obesity (46.8%), osteoarthritis (35.8%), hypertension (35.1%), heart disease (13.7%) and lung disease (11.9%).

**TABLE 1:** Baseline sociodemographic and behavioural characteristics of 2815 participants of the ELSA Study (2012/2013) stratified by serum 25(OH)D status.

	Total (*n* = 2815)	25(OH)D sufficiency (*n* = 1348)	25(OH)D insufficiency (*n* = 915)	25(OH)D deficiency (*n* = 552)
Age, years	68.3 ± 6.2	68.2 ± 5.8	68.3 ± 6.3	68.4 ± 6.7
Age, %				
60–69 years	62.8	63.3	62.2	62.3
70–79 years	31.7	32.0	32.0	30.8
80 years or more	5.5	4.7	5.8	6.9
Sex, %				
Female	52.2	51.5	49.9	57.6
Skin colour, %				
Non‐White	1.6	0.7	1.4	4.2* ^,^ ^†^
Marital status, %				
Without conjugal life	27.1	22.1	30.6*	33.5*
Education, %				
> 13 years	37.2	38.0	37.5	34.8
12–13 years	28.0	28.7	28.1	25.9
≤ 11 years	34.8	33.3	34.4	39.3
Wealth, %				
Highest quintile	29.0	32.3	27.8	22.6*
Fourth quintile	25.6	27.4	25.6	21.0*
Third quintile	21.8	22.2	20.5	23.2
Second quintile	14.3	12.0	15.2	18.5*
Lowest quintile	7.4	4.5	8.2ª	13.1* ^,^ ^†^
Not reported	1.9	1.6	2.7	1.6
Smoking, %				
Non‐smoker	38.8	40.7	38.7	34.4
Ex‐smoker	52.8	53.7	52.9	50.7
Smoker	8.4	5.6	8.4	14.9* ^,^ ^†^
Alcohol intake, %				
Rarely/never	14.8	11.8	15.7	20.8ª
Often	41.2	41.8	41.4	39.1
Daily	38.7	42.7	37.6	31.0*
Not reported	5.3	3.7	5.3	9.1* ^,^ ^†^
Physical activity, %				
Sedentary lifestyle	21.7	18.5	24.0*	25.9*

*Note*: Data are expressed as percentage, mean and standard deviation (SD).

* Significant difference from sufficiency, *p <* 0.05.

^†^ Significant difference from insufficiency, *p <* 0.05.

**TABLE 2:** Baseline clinical conditions and additional characteristics of 2815 participants of the ELSA Study (2012/2013) stratified by serum 25(OH)D status.

	Total (*n* = 2815)	25(OH)D sufficiency (*n* = 1348)	25(OH)D insufficiency (*n* = 915)	25(OH)D deficiency (*n* = 552)
Clinical conditions, %				
Hypertension	35.1	33.	35.3	38.8
Diabetes mellitus	7.7	7.0	7.9	9.2
Cancer	4.8	5.4	4.2	4.2
Heart disease	13.7	13.7	13.7	13.2
Lung disease	11.9	11.2	12.7	12.5
Stroke	2.5	2.1	2.3	3.6
Osteoporosis	6.4	9.2	3.8ª	4.0*
Osteoarthritis	35.8	35.5	35.6	37.1
Dementia	0.4	0.4	0.2	0.4
Hip fracture	0.2	0.2	0.3	0.2
Depressive symptoms	6.3	5.0	6.3	9.2*
Falls in previous year, %				
None	76.9	76.7	75.4	79.9
Single fall	15.7	15.5	16.8	14.5
Recurrent falls	7.4	7.8	7.8	5.6
Visual perception %				
Excellent/very good	54.6	56.2	53.7	52.4
Good	37.2	36.5	38.2	37.0
Fair/poor	8.2	7.3	8.1	10.6
Back pain, %				
No pain	88.8	89.5	87.2	89.7
Mild to moderate pain	8.8	8.3	10.1	8.0
Intense to severe pain	2.4	2.2	2.7	2.3
Lower limb pain, %				
No pain	82.3	83.2	81.0	82.1
Mild to moderate pain	12.5	12.0	13.2	12.7
Intense to severe pain	5.2	4.8	5.8	5.2
Anthropometry				
Waist circumference, cm	94.8 ± 12.8	93.0 ± 11.9	96.0 ± 13.1*	97.0 ± 13.7*
Abdominal obesity	46.8	40.9	49.5*	56.5*
Performance measures				
Grip strength, Kg	31.6 ± 10.4	31.8 ± 10.3	32.0 ± 10.5	30.5 ± 10.5
Dynapenia, %	5.5	4.7	5.3	7.8
Memory performance, score	11.4 ± 3.2	11.5 ± 3.1	11.3 ± 3.2	11.2 ± 3.4
Gait speed, m/s	1.1 ± 0.2	1.1 ± 0.2	1.1 ± 0.2	1.1 ± 0.2
Season – blood collection, %				
Spring	6.8	4.6	7.1	11.8* ^,^ ^†^
Summer	22.6	30.6	19.1*	8.9* ^,^ ^†^
Autumn	43.4	46.1	44.8	34.2* ^,^ ^†^
Winter	27.2	18.7	29.0*	45.1* ^,^ ^†^
Vitamin D supplementation	3.9	6.9	1.1*	1.3*
Carbamazepine	0.8	0.7	1.0	0.7

*Note*: Data are expressed as percentage, mean and standard deviation (SD).

* A significant difference from sufficiency, *p <* 0.05.

^†^ A significant difference from insufficiency, *p <* 0.05.

Compared with individuals with serum 25(OH)D sufficiency, those with insufficiency and deficiency were more likely to be living with a partner, were more often in the lowest wealth quintile, had a more sedentary lifestyle, a lower prevalence of osteoporosis, a higher prevalence of abdominal obesity, a greater proportion of blood samples collected in winter and lower usage of vitamin D supplementation. Individuals with serum 25(OH)D deficiency exhibited a higher frequency of non‐white skin colour, were current smokers, consumed less alcohol and experienced more depressive symptoms. In comparison with individuals having serum 25(OH)D insufficiency, those with serum 25(OH)D deficiency demonstrated a greater frequency of non‐white skin colour, were more frequently in the lowest wealth quintile, had a higher rate of smoking and showed a higher proportion of blood samples collected in winter.

Comparisons between included and excluded individuals revealed that excluded participants were older, lived without a partner, belonged to a lower wealth quintile, smoked more, consumed less alcohol daily and were more physically inactive. The excluded participants also exhibited a higher prevalence of hypertension, diabetes, cancer, heart disease, stroke, severe back pain and abdominal obesity than those included. Additionally, lower means of serum 25(OH)D levels, grip strength and gait speed were observed, along with poorer memory performance. Furthermore, a higher proportion of blood samples was collected from excluded individuals during the winter compared with included individuals (Tables S1 and S2).

The incidence density of slowness for 1000 person‐years during the six‐year follow‐up period was 67.4 (95% CI: 60.9–74.6) among those with serum 25(OH)D sufficiency, 76.7 (95% CI: 68.3–86.2) among those with serum 25(OH)D insufficiency, and 90.7 (95% CI: 78.5–104.9) among those with serum 25(OH)D deficiency (Table 3).

**TABLE 3:** Incidence density of slowness per 1000 person‐year by serum 25(OH) status, observed over six years of follow‐up ‐ ELSA Study (2012–2018).

Serum 25(OH)D level	Incidence density and 95% CI
Sufficiency	67.4 (60.93–74.64)
Insufficiency	76.7 (68.30–86.22)
Deficiency	90.7 (78.46–104.92)^a^

*Note*: Incidence density of slowness per 1000 person‐years.

^a^ Significant difference compared with serum 25(OH)D sufficiency according to a 95% CI.

Table 4 and Figure S1 summarise the results from the association between serum 25(OH)D status and the incidence of slowness. A significant association was identified between serum 25(OH)D deficiency and the incidence of slowness (IRR: 1.22; 95% CI: 1.01–1.49) in the fully adjusted model. Therefore, it can be estimated that individuals with serum 25(OH)D deficiency, compared with those with sufficient levels, have a 22% higher risk of developing slowness over the next six years, independent of their sociodemographic, behavioural and clinical characteristics. No significant association was identified between serum 25(OH)D insufficiency and the incidence of slowness (IRR: 1.10; 95% CI: 0.94–1.29).

**TABLE 4:** Results from the final adjusted Poisson regression model showing the incidence rate ratios (IRR) of slowness by serum 25(OH)D status over a six‐year of follow‐up ‐ ELSA Study (2012–2018).

Serum 25(OH)D level	IRR	95% CI
Sufficiency	1.00	
Insufficiency	1.10	(0.94–1.29)
Deficiency	1.22	(1.01–1.49)

*Note*: Values are presented by incidence rate ratio (IRR) and 95% confidence interval. Model controlled by sex, age, race, education, physical activity level, smoking, diabetes, heart disease, lung disease, osteoporosis, osteoarthritis, stroke, memory performance, dementia, cancer, abdominal obesity, dynapenia, depressive symptoms, visual perception, lower limb pain when walking, back pain when walking, falls, hip fracture, season of blood collection, use of carbamazepine and vitamin D supplementation.

## 4 DISCUSSION

In this large sample of older English individuals, we found that serum 25(OH)D deficiency is a risk factor for the incidence of slowness.

Cross‐sectional studies have demonstrated an association between low serum 25(OH)D levels and slower gait speed in older individuals.^12^, ^13^, ^14^, ^15^ However, this association has not been confirmed in longitudinal studies. Analysing 988 individuals aged ≥77 years, Houston and collaborators found no link between serum 25(OH)D deficiency (<20 ng/mL, which corresponds to <50 nmol/L) and gait speed decline on the 3‐m walk test at the three‐year follow‐up.^16^ Similarly, Houston and collaborators observed no association between baseline serum 25(OH)D levels, considered a continuous variable, and changes in gait speed on the 400‐m walk test at the 12‐month follow‐up involving 368 individuals aged 70 to 89. Furthermore, changes in serum 25(OH)D status over time were not linked with gait speed improvements.^17^ In contrast, a baseline association was found between serum 25(OH)D deficiency (<20 ng/mL, equivalent to <50 nmol/L) and slow gait speed on the 20‐m and 400‐m walk tests in 2641 individuals aged 71 to 80. Nevertheless, the decline in gait speed remained significant only on the 400‐m walk test after a four‐year follow‐up assessment.^18^

Methodological differences may explain the conflicting results between these studies and ours. Unlike our study, which classified serum 25(OH)D levels according to the cut‐off of the US Institute of Medicine, the aforementioned studies were based on The Endocrine Society cutoff: sufficiency (≥75 nmol/L), insufficiency (50 to <75 nmol/L) and deficiency (<50 nmol/L).^36^ Higher cut‐offs may be less sensitive to functional outcomes like mobility limitations. Notably, the gait speed test distances differed. Despite assessing mobility limitation, the 400‐m walk test is more complex, as it also evaluates cardiorespiratory performance.^37^, ^38^ Therefore, the significant results for this test alone in the study by Houston and collaborators may reflect that cardiorespiratory fitness declines before mobility over four years. Moreover, the samples in the other cited studies comprised older individuals who may already have a lower baseline gait speed, leaving less mobility reserve to decline over time. Additionally, the studies referenced had shorter follow‐up periods, which may not be sufficient to observe a significant gait speed decline. Lastly, none excluded individuals with slowness at baseline, which hampers the determination of the association between serum 25(OH)D levels and the incidence of slowness.

Our study goes a step further by reinforcing that the harm caused by serum 25(OH)D deficiency goes beyond the known effects in osteomineral metabolism. It also impairs the mobility of older individuals, including those without any previous impairment, and can lead to slower gait speed, regardless of health conditions and individual particularities.

A potential mechanism for our findings is the extraosseous role of 25(OH)D in the musculoskeletal system. Through the genomic pathway, the active form of vitamin D binds to nuclear VDRs in muscle cells, triggering the synthesis of essential proteins for muscle metabolism.^39^, ^40^, ^41^, ^42^ Through the non‐genomic pathway, the active form of vitamin D binds to the cytoplasmic VDRs of muscle cells, activating signalling pathways to influx Ca^2+^ into the sarcoplasmic reticulum for muscle contraction.^39^, ^40^, ^41^, ^42^ In addition, VDRs present in satellite cells also activate signalling pathways for muscle fibre repair.^43^

Consequently, serum 25(OH)D deficiency disrupts these pathways, hindering the metabolism of muscle fibres, including contraction and repair capacity. Collectively, these mechanisms can result in muscle atrophy and diminished muscle strength function,^41^, ^44^ which can impair muscle performance during gait. Over time, it may lead to decreased gait speed, potentially resulting in mobility limitations, functional disability and an increased risk of falls.^23^, ^45^, ^46^, ^47^

Aside from its impact on muscle strength, serum 25(OH)D deficiency can reduce the physiological reserves of vital systems, including the immune, cardiovascular, respiratory and neural systems.^8^, ^48^, ^49^ This may contribute to the development of disabling clinical conditions like diabetes, cancer, cognitive decline and cardiovascular disease, which are closely associated with a reduction in gait speed among older individuals.^8^, ^48^, ^49^

Our study has several strengths and limitations. Among the strengths is that we used a large and representative sample of older individuals, and the wide range of socioeconomic, behavioural and clinical variables allowed robust adjustments to our regression models. Moreover, including three waves of the ELSA Study enabled a long follow‐up period.

However, there are limitations to consider. The ELSA Study only involved community‐dwelling individuals, limiting the generalizability of our findings to institutionalized individuals who may experience more significant mobility limitations. Although small, follow‐up losses were an inevitable source of bias in longitudinal studies. The ELSA Study did not include some important control variables for our models, such as parathormone (PTH) and creatinine. PTH is elevated in serum 25(OH)D deficiency (secondary hyperparathyroidism), which can reduce muscle strength. High creatinine levels indicate renal insufficiency, which can impair the 25(OH)D metabolism. The lack of nutritional data does not allow the investigation of dietary sources of vitamin D. Furthermore, although the exclusion of individuals with missing data at baseline could introduce a potential source of bias, it did not prevent us from obtaining significant results. Finally, blood samples were collected more frequently in winter, when there is a lower incidence of UVB rays and reduced cutaneous synthesis of vitamin D, which may have augmented the proportion of individuals with serum 25(OH)D deficiency.

Despite our innovative results, additional studies are needed to determine the long‐term trajectories of gait speed decline based on serum 25(OH)D status, incorporating the abovementioned issues that could not be addressed in our study.

## 5 CONCLUSION

Serum 25(OH)D deficiency was identified as a risk factor for the incidence of slowness in older individuals. These findings facilitate the early identification of individuals at a higher risk of slowness and enhance our understanding of the mechanisms underlying mobility decline. Given that slow gait speed is linked to an increased risk of functional dependence and adverse outcomes, monitoring vitamin D levels, particularly in older individuals, should be prioritised across various clinical settings and health services to minimise the complications associated with vitamin D deficiency, particularly in mobility.

## AUTHOR CONTRIBUTIONS

MML and TSA conceived and designed the study. TSA, AS and CO acquired the data. MML, ROM and TSA prepared data consistency. MML and TSA analysed and interpreted the data. MML and TSA wrote the manuscript. MML, ROM, AFS, TBS, SSL, LCS, TBPS, AS, CO and TSA revised and approved the manuscript.

## FUNDING INFORMATION

The present study was funded by the Economic and Social Research Council [ES/T008822/11]. ELSA is funded by the National Institute on Ageing USA [R01AG017644], and governmental departments of the United Kingdom coordinated by the National Institute for Health and Care Research (NIHR). The present study was also funded by the Brazilian fostering agencies Coordination for the Advancement of Higher Education Personnel (CAPES) [001 for MML], São Paulo Research Foundation (FAPESP) [18/13917–3 for TSA], National Council of Scientific and Technological Development (CNPq) [303577/2020–7 and 305338/2023–4 for TSA], and Institutional Programme of Internationalization (CAPES‐PrInt) [88887.717097/2022–00 for MML]. The funders were not involved in the manuscript.

## CONFLICT OF INTEREST STATEMENT

None to declare.

### PEER REVIEW

The peer review history for this article is available at https://www.webofscience.com/api/gateway/wos/peer-review/10.1111/dom.16317.

## Supporting information


**Data S1.** Supporting Information.

## Data Availability

Data from the ELSA Study are available from the UK Data Service for researchers who meet the criteria for access to confidential data, under conditions of the End User License http://ukdataservice.ac.uk/media/455131/cd137-enduserlicence.pdf. The data can be accessed from: http://discover.ukdataservice.ac.uk/series/?sn=200011. Contact with the UK data service regarding access to the English Longitudinal Study of Ageing can be made through the website http://ukdataservice.ac.uk/help/get-in-touch.aspx, by phone +44 (0)1206 872143 or by email at help@ukdataservice.ac.uk.
