# Supplementary material for: Is serum 25‐hydroxyvitamin D deficiency a risk factor for the incidence of slow gait speed in older individuals? Evidence from the English longitudinal study of ageing
Source: Diabetes Obes Metab. 2025 Mar 13;27(6):3104–12. doi: 10.1111/dom.16317 (PMC12046445; doi:10.1111/dom.16317)
Supplement: Supplementary file 1 — Data S1. Supporting Information. [file DOM-27-3104-s001.docx]

**Supplemental Appendix**

**Table S1**. Sociodemographic and behavioural characteristics of individuals included and excluded from the analytical sample due to the lack of data on covariates at baseline (ELSA - 2012/13).

| **Variables** | **Included**  **(n = 2,815)** | **Excluded**  **(n = 1,232)** |
| --- | --- | --- |
| **Age, years** | 68.3 ± 6.1 | 69.3 ± 6.7^*^ |
| **Age, %** |  |  |
| 60 – 69 years | 62.8 | 57.6^*^ |
| 70 – 79 years | 31.8 | 34.7 |
| 80 years or more | 5.4 | 7.7^*^ |
| **Sex, %** |  |  |
| Female | 52.2 | 53.7 |
| **Skin colour, %** |  |  |
| Non-White | 1.6 | 2.0 |
| **Marital status, %** |  |  |
| Without conjugal life | 27.1 | 30.3^*^ |
| **Education%** |  |  |
| > 13 years | 37.2 | 33.7 |
| 12-13 years | 28.0 | 28.5 |
| ≤ 11 years | 34.8 | 37.8 |
| **Wealth, %** |  |  |
| Highest quintile | 28.9 | 29.1 |
| 4^th^ quintile | 25.6 | 23.2 |
| 3^rd^ quintile | 21.9 | 19.4 |
| 2^nd^ quintile | 14.3 | 15.6 |
| Lowest quintile | 7.4 | 11.0^*^ |
| Not reported | 1.9 | 1.7 |
| **Smoking, %** |  |  |
| Non-smoker | 38.8 | 36.3 |
| Ex-smoker | 52.8 | 53.8 |
| Smoker | 8.4 | 9.9^*^ |
| **Alcohol intake, %** |  |  |
| Rarely/never | 14.8 | 17.9 |
| Often | 41.2 | 36.3^*^ |
| Daily | 38.7 | 37.5 |
| Not reported | 5.3 | 8.4^*^ |
| **Physical activity, %** |  |  |
| Sedentary lifestyle | 21.7 | 29.5^*^ |

Note: Data are expressed as percentage, mean, and standard deviation (SD). ^*^ Significantly different from included (p <0.05).

**Table S2**. Clinical conditions and other characteristics of individuals included and excluded from the analytical sample due to the lack of data on covariates at baseline (ELSA - 2012/13).

| **Variables** | **Included**  **(n = 2,815)** | **Excluded**  **(n = 1,232)** |
| --- | --- | --- |
| **Clinical conditions, %** |  |  |
| Hypertension | 35.1 | 43.7^*^ |
| Diabetes mellitus | 7.7 | 11.4^*^ |
| Cancer | 4.8 | 7.5^*^ |
| Heart disease | 13.7 | 21.9^*^ |
| Lung disease | 11.9 | 13.2 |
| Stroke | 2.5 | 4.2^*^ |
| Osteoporosis | 6.4 | 5.8 |
| Osteoarthritis | 35.8 | 34.9 |
| Dementia | 0.4 | 0.2 |
| Falls in previous year, % |  |  |
| None | 76.9 | 76.2 |
| Single fall | 15.7 | 15.3 |
| Recurring falls | 7.4 | 8.5 |
| Hip fracture | 0.3 | 0.2 |
| Depressive symptoms, % | 6.3 | 6.8 |
| Visual perception, % |  |  |
| Excellent / very good | 54.6 | 51.3 |
| Good | 37.2 | 39.5 |
| Fair / poor | 8.2 | 9.2 |
| Back pain, % |  |  |
| No pain | 88.8 | 86.3 |
| Mild to moderate pain | 8.8 | 9.3 |
| Intense to severe pain | 2.4 | 4.4^*^ |
| Lower limb pain, % |  |  |
| No pain | 82.3 | 80.4 |
| Mild to moderate pain | 12.5 | 12.9 |
| Intense to severe pain | 5.2 | 6.7 |
| Serum 25(OH)D, nmol/L (mean ± SD) | 51.6 ± 23.1 | 46.9 ± 21.4^*^ |
| Serum 25(OH)D status, % |  |  |
| Sufficiency | 47.9 | 41.8 |
| Insufficiency | 32.5 | 33.0 |
| Deficiency | 19.6 | 25.2 |
| **Anthropometry** |  |  |
| Waist circumference, cm | 94.8 ± 12.8 | 96.7 ± 13.6^*^ |
| Abdominal obesity | 46.7 | 54.2^*^ |
| **Performance measures** |  |  |
| Grip strength, Kg | 31.6 ± 10.4 | 30.6 ± 10.4^*^ |
| Dynapenia, % | 5.5 | 6.9 |
| Memory performance, score | 11.4 ± 3.2 | 11.0 ± 3.3^*^ |
| Gait speed, m/s | 1.08 ± 0.2 | 1.06 ± 0.2^*^ |
| **Season – blood collection, %** |  |  |
| Spring | 6.8 | 7.3 |
| Summer | 22.6 | 11.3^*^ |
| Autumn | 43.4 | 41.4 |
| Winter | 27.2 | 40.0^*^ |
| **Vitamin D supplementation** | 3.9 | 3.4 |
| **Carbamazepine** | 0.8 | 1.6 |

Note: Data are expressed as percentage, mean, and standard deviation (SD). ^*^ Significantly different from included (p <0.05).

**Figure S1**. Fully adjusted Poisson regression model for the association between serum 25(OH)D status and incidence of slowness. Circles represent the incidence rate ratio (IRR) of slowness, and bars represent the 95% CI of the IRR.
